# Rapid ecological specialization despite constant population sizes

**DOI:** 10.7717/peerj.6476

**Published:** 2019-04-19

**Authors:** Andrinajoro R. Rakotoarivelo, Paul O’Donoghue, Michael W. Bruford, Yoshan Moodley

**Affiliations:** 1 Department of Zoology, University of Venda, Thohoyandou, Limpopo, Republic of South Africa; 2 Natiora Ahy, Antananarivo, Madagascar; 3 Specialist Wildlife Services, Specialist Wildlife Services, St Asaph, United Kingdom; 4 Cardiff School of Biosciences, Cardiff University, Cardiff, United Kingdom

**Keywords:** Bushbuck, Convergent evolution, Ecological adaptation, Species complex, Stable demography

## Abstract

**Background:**

The bushbuck, *Tragelaphus scriptus*, is a widespread and ecologically diverse ungulate species complex within the spiral-horned antelopes. This species was recently found to consist of two genetically divergent but monophyletic lineages, which are paraphyletic at mitochondrial (mt)DNA owing to an ancient interspecific hybridization event. The Scriptus lineage (*T. s. scriptus*) inhabits the north-western half of the African continent while Sylvaticus (*T. s. sylvaticus*) is found in the south-eastern half. Here we test hypotheses of historical demography and adaptation in bushbuck using a higher-resolution framework, with four nuclear (MGF, PRKCI, SPTBN, and THY) and three new mitochondrial markers (cytochrome b, 12S rRNA, and 16S rRNA).

**Methods:**

Genealogies were reconstructed for the mitochondrial and nuclear data sets, with the latter dated using fossil calibration points. We also inferred the demographic history of Scriptus and Sylvaticus using coalescent-based methods. To obtain an overview of the origins and ancestral colonisation routes of ancestral bushbuck sequences across geographic space, we conducted discrete Bayesian phylogeographic and statistical dispersal-vicariance analyses on our nuclear DNA data set.

**Results:**

Both nuclear DNA and mtDNA support previous findings of two genetically divergent Sylvaticus and Scriptus lineages. The three mtDNA loci confirmed 15 of the previously defined haplogroups, including those with convergent phenotypes. However, the nuclear tree showed less phylogenetic resolution at the more derived parts of the genealogy, possibly due to incomplete lineage sorting of the slower evolving nuclear genome. The only exception to this was the montane Menelik’s bushbuck (Sylvaticus) of the Ethiopian highlands, which formed a monophyletic group at three of four nuclear DNA loci. We dated the coalescence of the two lineages to a common ancestor ∼2.54 million years ago. Both marker sets revealed similar demographic histories of constant population size over time. We show that the bushbuck likely originated in East Africa, with Scriptus dispersing to colonise suitable habitats west of the African Rift and Sylvaticus radiating from east of the Rift into southern Africa via a series of mainly vicariance events.

**Discussion:**

Despite lower levels of genetic structure at nuclear loci, we confirmed the independent evolution of the Menelik’s bushbuck relative to the phenotypically similar montane bushbuck in East Africa, adding further weight to previous suggestions of convergent evolution within the bushbuck complex. Perhaps the most surprising result of our analysis was that both Scriptus and Sylvaticus populations remained relatively constant throughout the Pleistocene, which is remarkable given that this was a period of major climatic and tectonic change in Africa, and responsible for driving the evolution of much of the continent’s extant large mammalian diversity.

## Introduction

The bushbuck (*Tragelaphus scriptus*) is a well-known, highly diverse species complex of spiral-horned antelopes, inhabiting most of sub-Saharan Africa (Moodley et al., 2009; Hassanin et al., 2012). This species complex is unique, being the most widespread and ecologically diverse of any bovid species and occurring in approximately 73% of the total land area of sub-Saharan Africa. Across this vast and heterogeneous region, bushbuck can be found in most habitat types (Moodley & Bruford, 2007) from forested to xeric zones and ranging in altitude from sea-level to 4,000 m.

**Figure 1:**
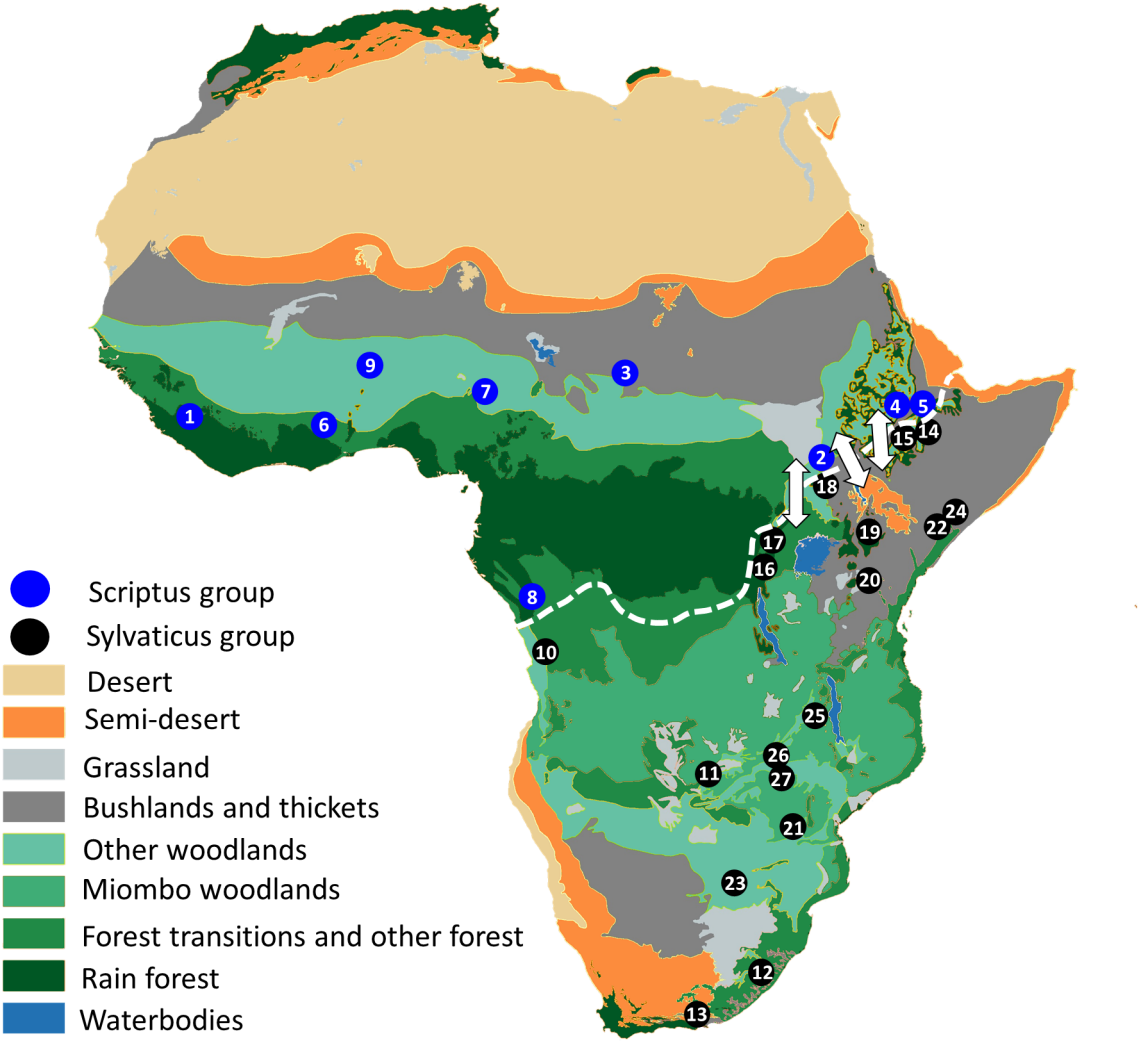
The land cover of Africa reconstructed from remotely sensed data (redrawn from Mayaux et al., 2004). The geographical distribution of sampling localities included in the present study are shown on the map. Taxa are plotted as dots and designated either blue for *Scriptus* or black for *Sylvaticus*. Samples are numbered according to Table 1. A dashed white line divides the distributions of both groups and white arrows show zones of potential gene flow.

Phenotypic diversity among bushbuck populations is unprecedented among the bovids. Although the number of subspecies described varies, up to twenty four have been recognised by a single author (Lydekker, 1914;  Allen, 1939). The complex can be subdivided into two divergent morphological groups which inhabit the western and northern (*T. s. scriptus* group) and eastern and southern (*T. s. sylvaticus* group) parts of the species range (Fig. 1), hereafter Scriptus and Sylvaticus for ease of reference. Scriptus is smaller and less dimorphic, but it possesses a heavily striped white harness-like pattern, whereas most populations of the larger Sylvaticus have little to no striping at all. Although known to favour areas of thick cover wherever they occur, bushbuck do not inhabit the dense rainforest of the Congo basin, preferring the mosaic landscapes at its fringe. The two groups are therefore separated in the west and south by the Lower Congo valley and the Congo basin respectively, but in eastern Africa Scriptus and Sylvaticus come into secondary contact from the northern end of the Albertine rift along the Imatong and Didinga Mountains of South Sudan following the rift into the Ethiopian Highlands (white arrows, Fig. 1). Within this zone of contact, the phenotypic integrity of each form may be maintained through habitat preference; the Scriptus form inhabits the low lands while the large, dark and heavy-coated Sylvaticus montane bushbuck inhabits the high altitude forests, although evidence of gene flow has been observed (Moodley & Bruford, 2007).

Scriptus and Sylvaticus can also be separated genetically. Initial mitochondrial (mt)DNA studies divided the bushbuck into Scriptus and Sylvaticus, but with either lineage more closely related to other *Tragelaphus* species than to each other (Moodley & Bruford, 2007; Moodley et al., 2009). This mtDNA paraphyly prompted some authors to regard Scriptus and Sylvaticus as independent species (Moodley et al., 2009; Hassanin et al., 2012;  Hassanin et al., 2018), possibly evolving through convergent evolution ( Wronski & Moodley, 2009). However, a recent analysis of nuclear DNA among the spiral-horned antelopes showed that Scriptus and Sylvaticus, although genetically divergent, are reciprocally monophyletic and so the bushbuck may henceforth be considered a single species. Paraphyletic Scriptus and Sylvaticus mtDNA lineages thus arose through an ancient interspecific hybridization event (Rakotoarivelo et al., in press).

Across its range, the bushbuck was highly structured into 23 phylogenetically distinct haplogroups (Scriptus 8; Sylvaticus 15), each with differing levels of ecological specialization. Among the more specialized haplogroups, the montane (*T. s. meneliki*, *T. s. powelli*, *T. s. barkeri* and *T. s. delamerei*), and more xeric-adapted (*T. s. decula*, *T. s. dodingae*, *T. s. fasciatus1*, *T. s. fasciatus2* and *T. s. roualeyni*) appear to have evolved more than once through convergent evolution (Moodley & Bruford, 2007). Much of the mtDNA variation in the complex is structured according to ecoregion (Olson et al., 2001), suggesting local ecological conditions as a driver for the evolution of specialization. Ecological conditions are in turn driven by a combination of local geology and an oscillating Pleistocene paleoclimate (Vrba, 1995; Bobe & Behrensmeyer, 2004; Hernandez Fernández & Vrba, 2005). However, where the species evolved and its subsequent routes of colonization and diversification are still unknown.

**Table 1:** Species-wide genetic sampling of bushbuck across sub-Saharan Africa.

	**Voucher**/ **Refenece**	**Sample**	**mtDNAHaplogroup**^a^	**TaxonomicSubspecies**^b^	**Common name**^b^	**Locality**	**Lat.**	**Long.**	**Country**	**Source**
1	20.7.10.21	scriptus_SL	*scriptus*	*scriptus*	Senegal bushbuck	Sierra Leone	7.54	−11.12	Sierra Leone	Natural History Museum, London
2	Uganda 368	dodingae1	*dodingae*	*dodingae*	Kidepo bushbuck	Kedef Valley, western Dodinga Hills	4.45	33.31	South Sudan	Powell Cotton Museum, Birchington, Kent
3	Chad 116	bor1	*bor*	*bor*	Nile bushbuck	Bouroum	10.45	18.8	Chad	Powell Cotton Museum, Birchington, Kent
4	AD2	decula2	*decula*	*decula*	Abyssinian bushbuck	Din Din	8.45	40.1	Ethiopia	Travel Ethiopia, Addis Ababa
5	AD1	decula1	*decula*	*decula*	Abyssinian bushbuck	Din Din	8.45	40.1	Ethiopia	Travel Ethiopia, Addis Ababa
6	GH4849	Lowervolta1	Lower Volta	*scriptus*	Lower Volta bushbuck	Ejura, Ashanti Region	7.38	−1.37	Ghana	Department of Evolutionary Biology, University of Copenhagen
7	26344	Niger1	Niger	*scriptus*	Niger bushbuck	Aningo	8.6	8.85	Nigeria	Nationaal Natuurhistorisch Museum, Leiden
8	17820	phaleratus1	*phaleratus*	*phaleratus*	Cabinda bushbuck	Tshimbali	−4.72	13.1	DRC	Royal Museum for Central Africa, Tervuren
9	GH6335	UpperVolta1	Upper Volta	*scriptus*	Upper Volta bushbuck	Kasana, Upper West Region	10.88	−1.99	Ghana	Department of Evolutionary Biology, University of Copenhagen
10	B14201	Angola1	Angola	*ornatus*	Angolan bushbuck	Lifune	−8.4	13.45	Angola	Staatliche Naturhistorische Sammlungen Dresden
11	Zimbabwe 07	ornatus1	*ornatus*	*ornatus*	Chobe bushbuck	Kazungula	−17.78	25.27	Zimbabwe	Bromley Game Skin Tannery, Harare, Zimbabwe
12	Reference 16	scriptus2	scriptus2	*sylvaticus*	South African bushbuck	South Africa	−30.64	29.29	South Africa	
13	ECape 04	sylvaticus1	*sylvaticus*	*sylvaticus*	South African bushbuck	Humansdorp, Eastern Cape	−34.02	24.77	South Africa	Taxidermy Africa, Humansdorp, South Africa
14	AbyssiniaII 30	meneliki1	*meneliki* 1	*meneliki*	Menelik’s bushbuck	Cure Rey, Arussi Mountains	7.05	39.42	Ethiopia	Powell Cotton Museum, Birchington, Kent
15	AbyssiniaII 56	meneliki2	*meneliki* 2	*meneliki*	Menelik’s bushbuck	Boare, Arussi Mountains	7.45	39.45	Ethiopia	Powell Cotton Museum, Birchington, Kent
16	Congo 329	dianae1	*dianae*	*dianae*	Ituri bushbuck	Kasindi	−0.04	29.71	DRC	Powell Cotton Museum, Birchington, Kent
17	Congo 159	dama1	*dama*	*dama*	Kavirondo bushbuck	Irumu	1.45	29.87	DRC	Powell Cotton Museum, Birchington, Kent
18	Sudan I 27	barkeri1	*barkeri*	*barkeri*	Barker’s bushbuck	Lomuleng, Imatong Mountains	3.95	33	South Sudan	Powell Cotton Museum, Birchington, Kent
19	Reference 10	scriptus1	*delamerei* 2	*delamerei*	Lord Delamere’s bushbuck	Kenya	−0.28	37.02	Kenya	
20	MM0555	haywoodi1	*delamerei* 1	*meruensis*	Lord Delamere’s bushbuck	Mount Meru	−3.23	36.75	Tanzania	Department of Evolutionary Biology, University of Copenhagen
21	Zimbabwe 10	massaicus1	*massaicus*	*massaicus*	Massai bushbuck	Chiredzi	−21	31.5	Zimbabwe	Bromley Game Skin Tannery, Harare, Zimbabwe
22	Jubaland 34	fasciatus1	*fasciatus* 1	*fasciatus*	Somali bushbuck	Mona Mofa Camp, Jubaland	0	42.12	Somalia	Powell Cotton Museum, Birchington, Kent
23	Limpopo 12	roualeyni1	*roualeyni*	*roualeyni*	Limpopo bushbuck	Thabazimbi	−24.6	27.4	South Africa	Nico van Rooyen Taxidermy, Rosslyn, South Africa
24	Jubaland 14	fasciatus2	*fasciatus* 2	*fasciatus*	Somali bushbuck	Mona Mofa Camp, Jubaland	0	42.12	Somalia	Powell Cotton Museum, Birchington, Kent
25	17001	Luangwa1	Luangwa	*ornatus*	Luangwa bushbuck	Msandile	−13.5	32.75	Zambia	Livingstone Museum, Livingstone, Zambia
26	Zimbabwe 17	Zambezi1	Zambezi1	*ornatus*	Zambezi bushbuck	Kanyemba	−15.7	30.32	Zimbabwe	Taxidermy Enterprises, Bulawayo, Zimbabwe
27	Zimbabwe 06	Zambezi2	Zambezi2	*ornatus*	Zambezi bushbuck	Mhangura	−16.9	30.15	Zimbabwe	Bromley Game Skin Tannery, Harare, Zimbabwe

**Notes.**

a After Moodley & Bruford (2007).

b After  Haltenorth (1963). Where no common name exists the dominant geographic feature of the area was used.

DRC Democratic Republic of the Congo

Despite the research potential of this system, only mtDNA data have been generated for this species to date. Not only is the mitochondrial genome a single locus, it is also maternally inherited so mtDNA structure may not be representative of nuclear DNA structure in species with sex biases in dispersal/philopatry. Genetic drift is also more effective in sorting non-segregating mtDNA lineages as their effective population size is approximately four times smaller than segregating nuclear DNA. Therefore, whether the nuclear genome is structured similarly, or even whether Scriptus and Sylvaticus constitute different nuclear lineages, is unknown. Furthermore, demographic analyses that may evidence population responses to paleo-environmental conditions and a spatially-informed phylogeographic analysis of origins and colonisation routes have never been carried out.

To test the hypotheses of variation, structure and potential adaptation purported by previous mtDNA work, we sequenced representative bushbuck from across the species range using a higher-resolution multilocus framework of four nuclear introns, complemented by three further mtDNA markers. We further reconstructed both the demographic and phylogeographic histories of the bushbuck complex using this new data set to shed further light on the evolution of this species.

## Materials & Methods

### Taxon sampling

A total of 27 bushbuck individuals (excluding outgroups) were included in this study. Samples sourced previously by Moodley & Bruford (2007) were re-extracted and representatives of all 23 mtDNA haplogroups were selected (Fig. 1; Table 1). As outgroups, we used both the distantly related *Bos taurus* as well as the most closely related lesser kudu (*Tragelaphus imberbis*) to root trees in several of the phylogenetic analyses.

### DNA sequencing

Four nuclear intron DNA markers (MGF—mast cell growth factor, PRKCI—protein-kinase CI, B-spectrin non-erythrocytic 1—SPTBN, and THY—thyrotropin) were amplified and sequenced in the 27 individuals above using previously published primers and methodology (Matthee et al., 2001). Additionally mtDNA sequences were amplified and sequenced from three mtDNA cytochrome b (Cyt b), 12S rRNA, and 16S rRNA (for mtDNA PCR and primer details see Arnason, Gullberg & Widegren, 1993; Simonsen, Siegismund & Arctander, 1998). In order for downstream comparison of summary statistics, the same individuals were sequenced for each locus. Sequences from each gene were first aligned using ClustalW (Thompson, Higgins & Gibson, 1994) as implemented in BioEdit (Hall, 1999), using default settings and thereafter manually to optimize homology. All heterozygous sites in the nuclear DNA were coded using the appropriate IUB code. Model selection for the best fitting substitution model for each gene was conducted in jModelTest (Posada, 2008; Darriba et al., 2012) under the Bayesian information criterion, which was preferred over the Akaike information criterion, to guard against over parameterization by averaging the likelihood over all included parameters.

### Analysis of genetic diversity and positive selection

The number of variable sites, number of parsimony informative sites and nucleotide frequencies were estimated for both mtDNA and nuclear DNA separately in MEGA 7 (Kumar, Stecher & Tamura, 2016). Further, for each locus we calculated standard diversity statistics in DnaSP 5.0 (Librado & Rozas, 2009). These included: the number of polymorphic sites (s), number of haplotypes, haplotype diversity (Hd), nucleotide diversity (*π*), and average number of pairwise differences per sequence (k). Summary statistics were also calculated for the total data and for each major clade inferred form phylogenetic analyses.

We used several analyses to test each of our seven loci for neutrality. The McDonald and Kreitman test (MKT) was used to detect signatures of selection and measure the amount of adaptive evolution within a species at the molecular level. Under this test, a neutrality index (NI) quantifies the direction of departure from neutrality, comparing the ratio of non-synonymous to synonymous variation between species (Dn/Ds) with the ratio of non-synonymous to synonymous variation within species (Pn/Ps). NI was calculated using the Standard and Generalized McDonald-Kreitman Test (MKT; Egea, Casillas & Barbadilla, 2008) website. Because silent mutations are neutral, a neutrality index lower than 1 (i.e., NI <1) indicates an excess of non-silent divergence, which occurs when positive selection is at work in the population. When positive selection is acting on the species, natural selection favors a specific phenotype over other phenotypes, and the favored phenotype begins to go to fixation in the species as the allele frequency for that phenotype increases (Biswas & Akey, 2006). Furthermore, we used the coalescent parameters Tajima’s D ( Tajima, 1989) and Fu’s Fs (Fu, 1997) to test for departures from the neutral theory and these were calculated in DnaSP v5.

### Phylogenetic analyses

Phylogenetic reconstruction was performed using both maximum likelihood (ML) and Bayesian approaches using the software Garli 2.0 (Zwickl, 2006) and BEAST v2.4.5 (Bouckaert et al., 2014) respectively. The total data matrix was partitioned by gene, with the parameters of nucleotide substitution models (12S rRNA −HKY + I + G, 16S −HKY, Cyt b- HKY + I, MGF −TIM1 + I, PRKCI −HKY, SPTBN −HKY, THY −TIM1ef + I) and unlinked across partitions. Each ML analysis was initiated from a random starting tree, with nodal support assessed using 1,000 bootstrap replicates. A 50% majority rule consensus tree was constructed using the CONSENSE program in the PHYLIP package (Felsenstein, 2005). Using BEAST, five independent runs of 1 billion generations each were performed; each run consisted of four Monte Carlo Markov chains (MCMC), with topologies sampled every 100,000 generations. The program Tracer 1.6 (Rambaut et al., 2014) was used to determine that the effective sample size (ESS) had reached >200 for all parameters. In each simulation the first 20% of generations were discarded as burn-in. Genealogies were also reconstructed for the nuclear and mitochondrial data sets and for each gene independently using the same MCMC parameters.

### Molecular dating

We dated our nuclear phylogeny, since the mtDNA of bushbuck are paraphyletically related (Moodley et al., 2009), and so mitochondrial branch lengths may be upwardly biased. Multiple fossil calibration points were used to scale nodal depth estimation. We calibrated the bushbuck divergence based on the earliest appearance of *T. scriptus* s.l. in the fossil record in Kenya (Leake & Harris, 2003) and Ethiopia (Kalb et al., 1982) as early as 3.9 Mya and a minimum age of constraint of 2.58 Mya as suggested by  Hassanin & Douzery (1999). An exponential distribution was used with a 2.5% probability quantile set at the age of the fossil with hard bound at the youngest bound and a soft maximum bound, beyond which it is unlikely that the divergence actually occurred. Our last calibration point constrained the evolution of the tribe Tragelaphinii 5.72 Mya (95% probability, 4.7–6.7 Mya; Deino et al., 2002). In the latter case, a normal distribution was used allowing for the actual node age to be equally younger or older than the fossil record. Phylogenetic relationships and divergence times were estimated using an uncorrelated relaxed lognormal Bayesian molecular clock approach in BEAST v. 2.4.5 software (Bouckaert et al., 2014). A Yule speciation process was applied to the tree inference through the MCMC (Markov chain Monte Carlo) with a random starting tree. All other parameters were the same as in previous analysis.

### Inferring historical demography

In addition to Tajima’s D and Fu’s Fs, which may be used to infer demography in neutrally evolving loci, demographic changes in both clades were also inferred from the observed mismatch distribution for each of the populations, calculating the raggedness index (R2) according to the population expansion model in DnaSP (Librado & Rozas, 2009). This measure quantifies the smoothness of the observed mismatch distribution, with lower raggedness characterizing a population that experienced a sudden expansion, whereas higher raggedness values suggest stationary or bottlenecked populations (Harpending et al., 1993; Harpending, 1994). Lastly, changes in effective population size were inferred using Bayesian Skyline Plots (BSP: Drummond et al., 2005). These plots utilize the coalescent properties of gene trees to plot population size changes over time, however, the inferred population sizes could potentially be biased downwards (population decline) if the sample set is significantly genetically structured (Ho & Shapiro, 2011;  Heller, Chikhi & Siegismund, 2013). To account for biases due to genetic structure, we divided the data into Scriptus and Sylvaticus groups and reconstructed their demographic histories separately using BEAST (Bouckaert et al., 2014). In order to incorporate stochastic differences between gene genealogies in the estimation of population parameters, we constructed multi-locus Extended Bayesian Skyline Plots (EBSP; Heled & Drummond, 2008) for each clade. In addition, EBSP estimates posterior probabilities for the number of population size change events. A mitochondrial divergence rate of 0.056 per million years was used ( Arbogast & Slowinski, 1998) as well as appropriate inheritance scalars were used to account for potential difference in effective population size between mtDNA and nuclear DNA. The lengths of the MCMC chains were set to 1 billion to achieve effective sample sizes (ESS) and proper mixing of Markov chains.

### Bayesian phylogeographic reconstruction

We attempted to reconstruct the phylogeographic history of two major clades of the bushbuck complex using our nuclear DNA data set. To do this, we employed the spatial diffusion approach under a Bayesian discrete phylogeographic framework in BEAST 1.8.4 (Lemey et al., 2009; Drummond et al., 2012). Five independent runs of 1 billion generations each were performed; each run consisted of four Monte Carlo Markov chains (MCMC), with topologies sampled every 100,000 generations. We used three geographical states corresponding to the continental regions where both lineages are present: west (W), east (E), and south (S). These phylogeographic analyses were run under a constant-size coalescent model, with molecular clock parameterised as described above and with a random starting tree as tree model. Bayesian Stochastic Search Variable Selection (BSSVS) was used to identify those rates (colonization routes) that were frequently invoked to explain the diffusion process (Lemey et al., 2009). The maximum clade credibility (MCC) tree was computed and annotated using the BEAST module TreeAnnotator v1.8.4 (Drummond et al., 2012). We then used SpreaD3 v0.9.6 (Bielejec et al., 2016; https://github.com/phylogeography/SpreaD3) to analyze and visualize the spatial diffusion incorporated in our Bayesian phylogeographic reconstruction. This was done by mapping the location—annotated MCC tree with the 95% highest posterior density (HPD) of node locations which was then export as a keyhole markup language (KML) file for animation of the spatial diffusion in virtual globe software. The final results were overlaid onto a base map of Africa. We also ran a further ancestral reconstruction analyses on our nuclear DNA data set using a statistical dispersal-vicariance model (S-DIVA) in the package RASP 4.0 (Reconstruct Ancestral State in Phylogenies; Yu, Harris & He, 2010; Yu et al., 2015). In this analysis we used the same West, East and South geographical regions and loaded the posterior sample of 10,001 trees previously produced in BEAST v. 2.4.5 software (Bouckaert et al., 2014). Only the most likely reconstruction was considered for each node.

### Genetic variation and its relationship to taxonomy and biogeography

To test whether nuclear DNA supported the hypothesis that ecology has driven genetic diversification in this complex (Moodley & Bruford, 2007), we tested the fit of a comprehensive biogeographic model (Olson et al., 2001) to the nuclear DNA data, relative to that of taxonomic and geographic models using a multiple regression on genetic distance matrices (MRM), implemented in DISTLM (Anderson, 2004). MRM involves a multiple regression of a response matrix on any number of explanatory matrices, where each matrix contains distances or similarities. Pair-wise genetic distances of nuclear DNA data between all 27 samples was used as the response matrix. The MRM method also allows the use of covariables to assess a models conditional effect on that of explanatory matrices. We defined the basic units for the taxonomy model relative to the proposed phenotypic classification of the bushbuck based on the combined classifications of Grubb-Best (Best, 1962; Grubb, 1985) used in Moodley & Bruford (2007) and also the recently published scheme of Groves & Grubb (2011). It should be note that the regression tests employed here test the taxonomic partitions in the data, and not whether these partitions comprise species-, subspecies- or population-level entities. A matrix of geographic coordinates (latitude and longitude) was included as a covariable to assess the possible the effect of isolation-by-distance (IBD) on the model being tested. In a wide-ranging species, IBD may significantly influence genetic structure due to the geographic distance separating the widely distributed sampling locations. MRM method allows the quantification of this effect, conditional on that of biogeography and taxonomy.

## Results

This study generated a total DNA sequence alignment of 4,676 bp, of which ingroup taxa accounted for 353 segregating sites. Nuclear introns were less diverse (2,596 bp, 26 segregating sites) than mitochondrial genes (2,080 bp, 353 segregating sites, see Table 2). All DNA sequences were found to be evolving neutrally (MKT: *χ*2 *P* > 0.1).

**Table 2:** Genetic diversity for mtDNA regions, nucDNA regions for all ingroup sequences and the two major *Scriptus* and *Sylvaticus* clades.

	**Locus**	**n**	**Size (bp)**	**S**	*π*	**h**	**Hd**	**k**	**S/k**
Entire species complex	12SrRNA	27	593	63	0.036	21	0.98	21.348	2.951
	16SrRNA	27	347	35	0.038	17	0.954	13.137	2.664
	Cytochrome*b*	27	1,140	255	0.072	24	0.991	82	3.11
	MGF	27	671	10	0.003	5	0.635	1.852	5.399
	PRCK1	27	498	2	0.0003	3	0.145	0.148	13.51
	SPTBN1	27	764	12	0.001	7	0.456	0.957	12.539
	THY	27	663	2	0.0008	3	0.501	0.541	3.696
*Scriptus* clade	12SrRNA	27	593	17	0.012	8	0.972	7.167	2.371
	16SrRNA	27	347	3	0.003	3	0.667	1	3
	Cytochrome*b*	27	1,140	90	0.028	8	0.972	32.389	2.778
	MGF	27	671	0	0	1	0	0	2.712
	PRCK1	27	498	2	0.001	3	0.556	0.611	0
	SPTBN1	27	764	0	0	1	0	0	3.273
	THY	27	663	0	0	1	0	0	0
*Sylvaticus* clade	12SrRNA	27	593	27	0.01	13	0.961	5.81	4.64
	16SrRNA	27	347	23	0.02	14	0.974	6.843	3.361
	Cytochrome*b*	27	1,140	158	0.035	16	0.987	40.333	3.917
	MGF	27	671	10	0.002	4	0.399	1.601	6.246
	PRCK1	27	498	0	0	1	0	0	0
	SPTBN1	27	764	13	0.002	7	0.634	1.542	9.155
	THY	27	663	1	0.0003	2	0.209	0.209	4.785

**Notes.**

S number of polymorphic sites *π* nucleotide diversity h number of haplotypes Hd haplotype diversity k average number of nucleotide differences S/k expansion coefficient

Statistically significant results were indicated by asterisks: *P < 0.05, **P < 0.01.

### Structure and divergence

**Figure 2:**
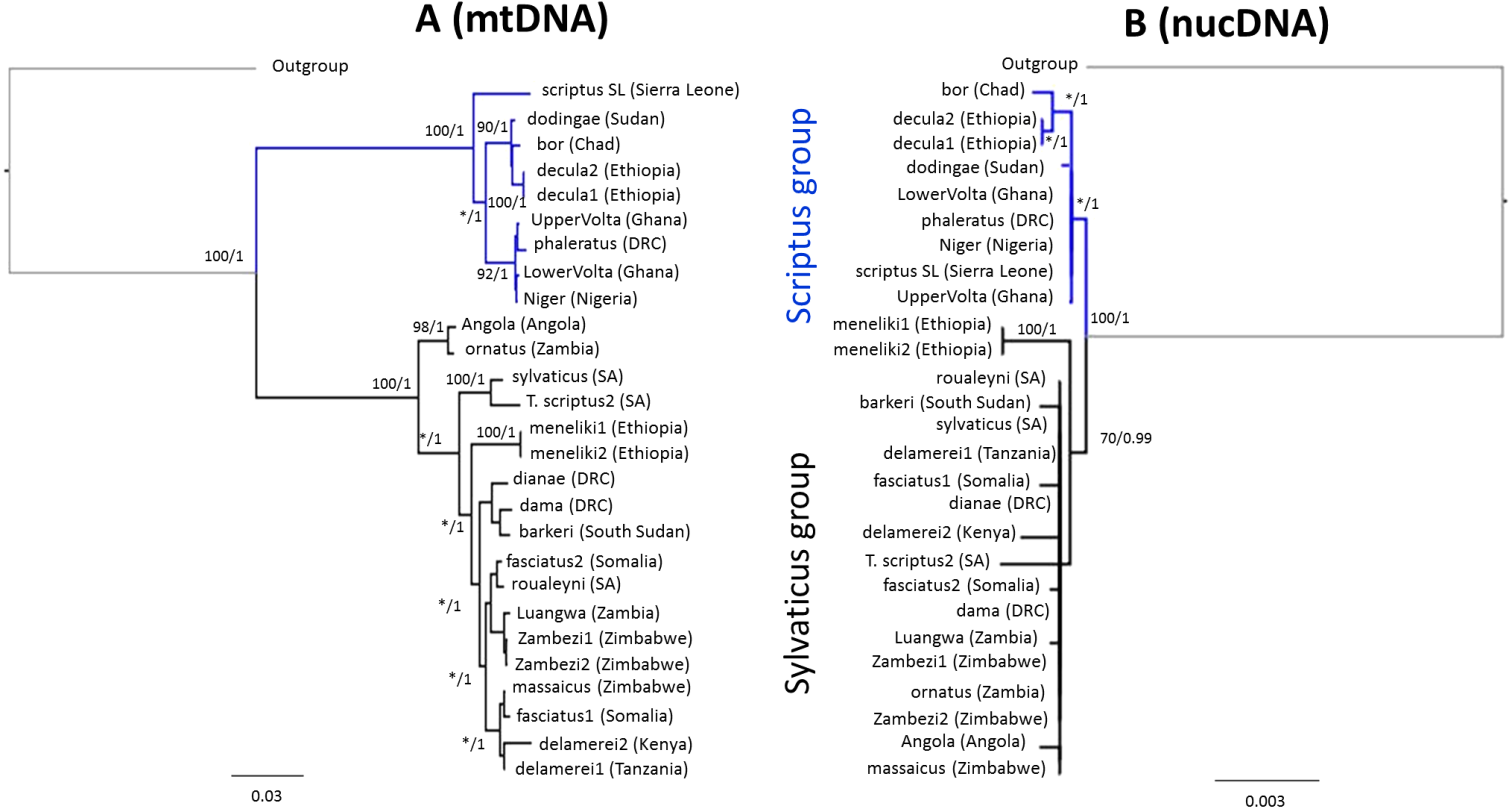
Tree topologies based on maximum likelihood retrieved from (A) the combined mtDNA data and (B) the combined nucDNA data. Values given above the branches represent maximum likelihood bootstrap values and maximum clade probabilities.

Phylogenetic analyses of mitochondrial (Fig. 2A) and nuclear (Fig. 2B) multilocus alignments yielded highly concordant ML topologies. Both marker sets recovered two well supported Scriptus and Sylvaticus lineages, although the level of phylogenetic resolution was much higher for mtDNA, recovering the general topology originally observed by Moodley & Bruford (2007), despite much smaller sample sizes. By contrast nuclear introns identified the lineage of the Kidepo bushbuck (*T. s. dodingae*) as well as a Nile-Abyssinian (*T. s. bor-T. s. decula*) bushbuck clade within Scriptus*.* The Sylvaticus clade was also less structured, with the montane Menelik‘s bushbuck (*T. s. meneliki*) being ancestral and the only resolvable clade. However, montane *T. s. barkeri* and *T. s. delamerei*, both lineages of the xeric–zone Somali bushbuck (*T. s. fasciatus*), as well as Luangwa and Angolan bushbuck lineages were characterized by higher nuclear divergence (Fig. 2B).

**Figure 3:**
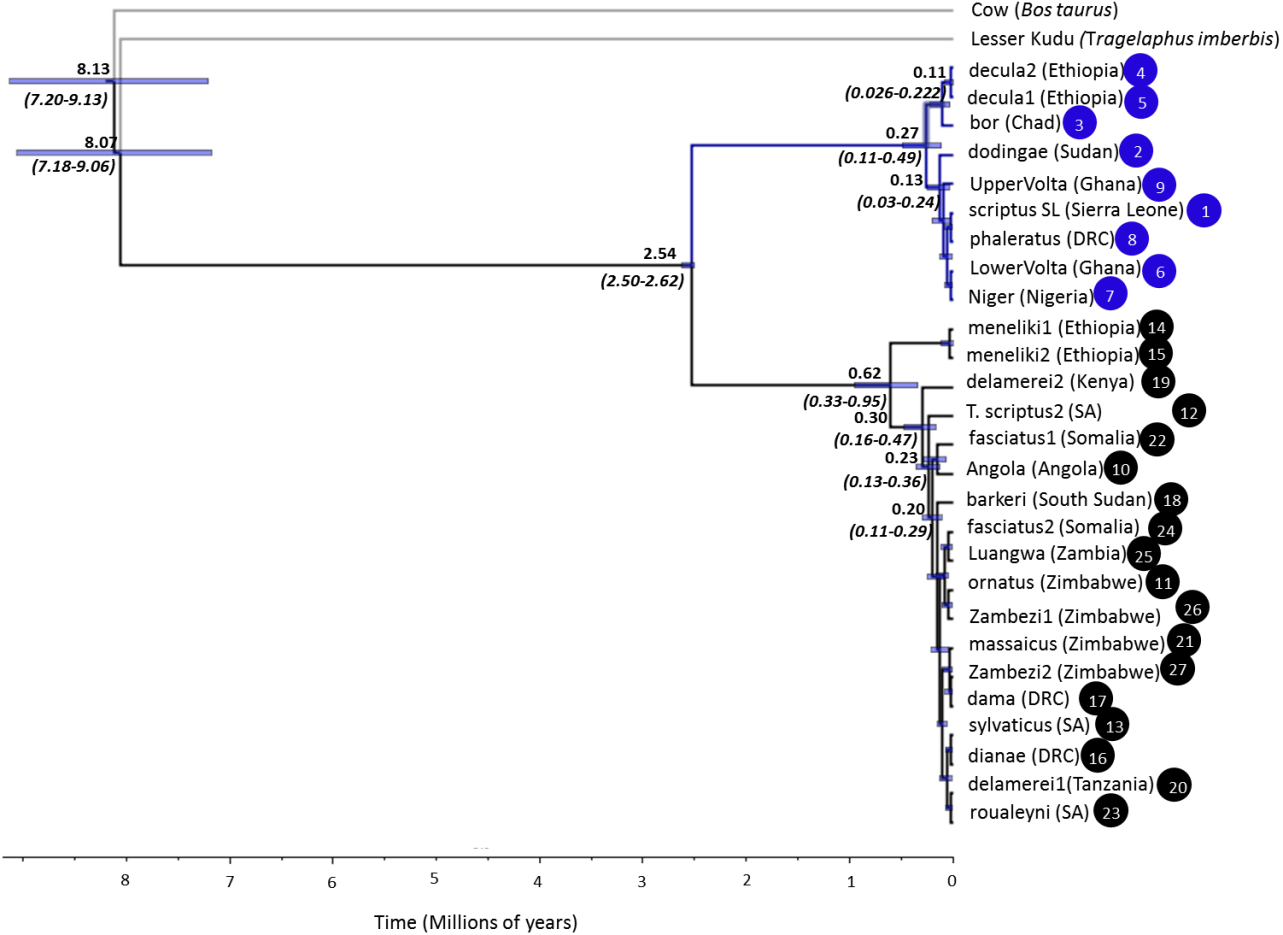
Multilocus Bayesian phylogeny of 27 bushbuck (Scriptus and Sylvaticus) individuals at four nuclear introns (MGF, PRKCI, SPTBN, and THY) reconstructed in BEAST. Median divergence time estimates (in MYA) are given for nodes appearing in more than 50% of the post-burn in posterior distribution. Nodal 95% HPD values are adjacent to their respective nodes and shown graphically by purple nodal bars. The two bushbuck lineages Scriptus and Sylvaticus are colored as in Fig. 1.

Bayesian dating of nuclear DNA loci estimated the coalescence of all ingroup gene tree lineages to the late Pliocene-early Pleistocene 2.5–2.62 Mya (95% HPD, Fig. 3). Divergence within each group occurred relatively recently in the Late Pleistocene. Scriptus lineages coalesced between 0.10–0.48 Mya (95% HPD) and the Nile-Abyssinian bushbuck clade to 0.03–0.22 Mya (95% HPD). Divergence within Sylvaticus was slightly earlier between 0.33–0.95 Mya (95% HPD) and 0.16–0.47 Mya (95% HPD) for non-Menelik’s bushbuck lineages.

### Demographic analyses

**Figure 4:**
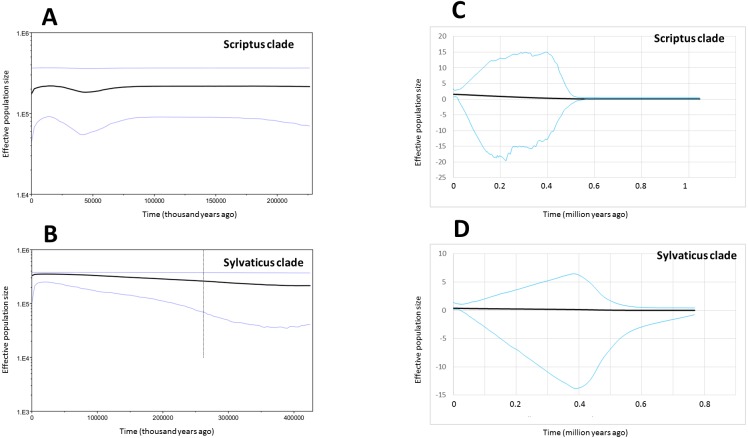
Bayesian Skyline Plots (BSPs) and Extended Bayesian Skyline Plots (EBSPs). (A-B) BSPs represent population size changes over time, inferred with mtDNA and an assumed divergence rate of 0.056 per million years. The *X*-axes are time in thousands of years. *Y*-axes are mean effective population sizes log-scale. Solid black lines represent median height and areas between blue lines encompass the 95% highest posterior density (HPD). (C-D) EBSPs represent population size changes over time in two of the mtDNA clades, inferred by mtDNA and nDNA. *X*-axes are time in millions of years, *Y*-axes are effective population size divided by generation time.

We found both Fu’s Fs and Tajima’s D indices to be slightly negative among nuclear and mitochondrial loci, for both Scriptus and Sylvaticus (Table 3). However, only locus SPTBN1 returned statistically significant indices, allowing a rejection of the neutrality/constant population size null hypothesis at the species level. Furthermore, the frequencies of pair-wise differences within each population were also consistent with a null hypothesis of constant population size, with non-significant raggedness indices (R2) for all mismatch distributions (Table 3). Additionally, both the single locus Bayesian skyline analyses based on mtDNA (Figs. 4A–4B) and the multilocus extended Bayesian skyline analyses of nuclear introns (Figs. 4C–4D) indicated that the effective population sizes of both Scriptus and Sylvaticus have remained relatively stable throughout the Pleistocene.

**Table 3:** Demography and tests of the neutral model for mtDNA regions , nDNA regions, and defined major clades of Bushbuck.

	**Locus**	**Fu’s Fs**	**Tajima’s D**	**Raggedness(R2)**	**Mismatch distribution**	**Tau (*τ*)**
Entire species complex	12SrRNA	−2.04	1.02	0.163	Multimodal	5.154
	16SrRNA	−1.007	1.244	0.185	Multimodal	5.302
	Cytochrome *b*	0.074	0.606	0.153	Multimodal	33.927
	MGF	0.93	−1.15678	0.107	Multimodal	0.607
	PRCK1	−2.223	−1.511	0.131	Unimodal	0.148
	SPTBN1	−3.091^*^	−2.312^**^	0.088	Unimodal	0
	THY	0.15	0.091	0.135	Unimodal	
*Scriptus* clade	12SrRNA	−1.788	0.401	0.186	Multimodal	4.105
	16SrRNA	−0.707	−0.359	0.229	Unimodal	1
	Cytochrome *b*	1.138	−0.113	0.17	Multimodal	13.51
	MGF	–	–	–	–	–
	PRCK1	−0.532	−0.583	0.185	Unimodal	0.611
	SPTBN1	–	–	–	–	–
	THY	–	–	–	–	–
*Sylvaticus* clade	12SrRNA	−3.842	−1.036	0.097	Multimodal	3.057
	16SrRNA	−4.371	−0.076	0.146	Multimodal	4.327
	Cytochrome *b*	−0.382	−0.562	0.113	Multimodal	22.63
	MGF	1.007	−1.618	0.106	Multimodal	0
	PRCK1	–	–	–	–	–
	SPTBN1	−2.257	−2.207^**^	0.1	Unimodal	0.303
	THY	−0.011	−0.529	0.104	Unimodal	0.209

Statistically significant results were indicated by asterisks: *P < 0.05, **P < 0.01.

### Bayesian phylogeographic reconstruction

**Figure 5:**
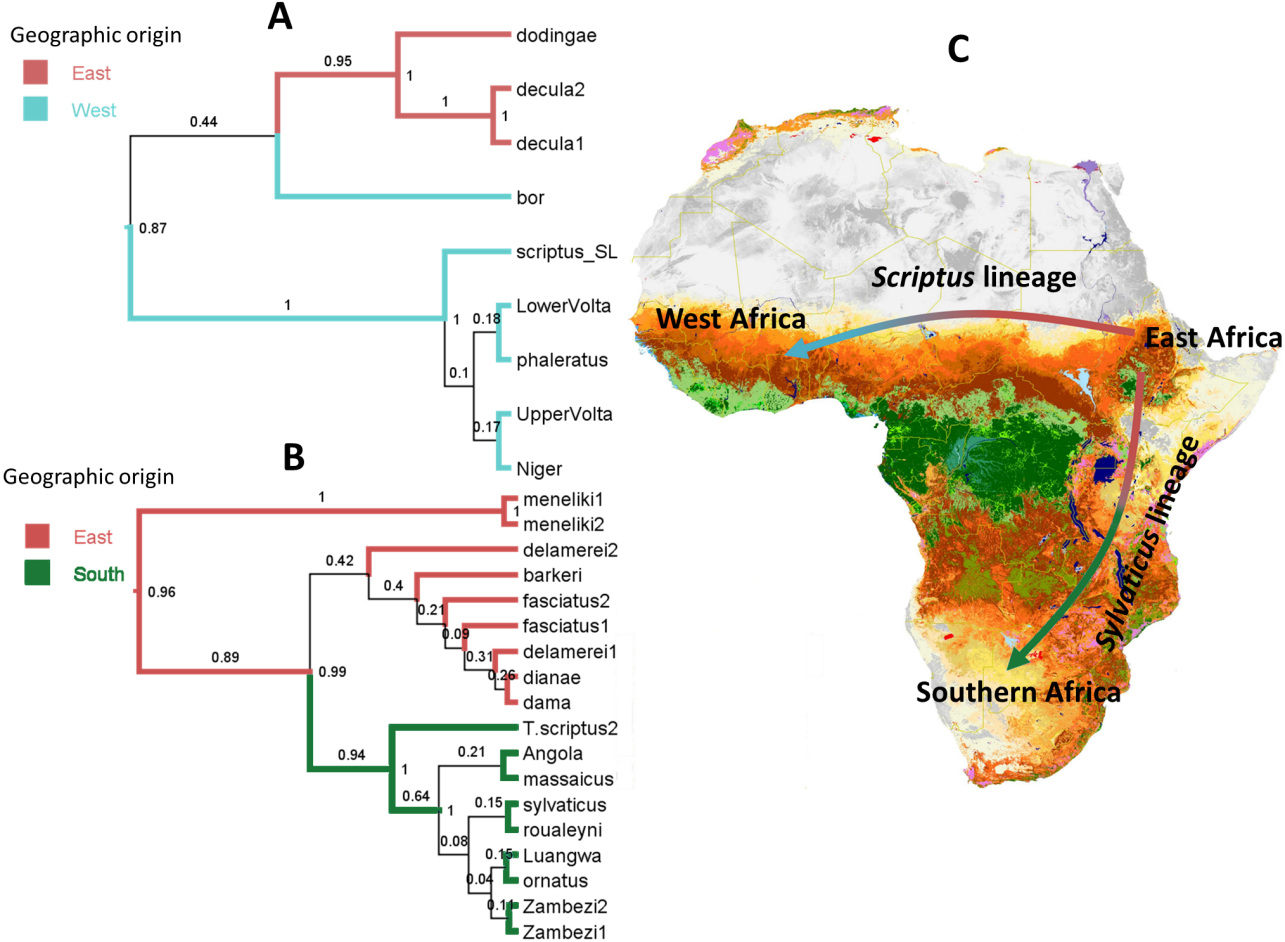
Bayesian ancestral range reconstruction and colonization history of bushbuck based on nDNA markers. (A) *Scriptus* lineage, (B) *Sylvaticus* lineage. (C) Colonization routes ofbushbuck species complex identified by BSSVS. Lines between geographic regions represent branches in the MCC tree along which the relevant location transition occurs. Numbers above branches are Bayesian posterior probabilities (PP). The coloured branch lengths represent the ancestral range with highest marginal probability for each lineage as inferred in BEAST (only branches with *PP* > 0.5). Numbers at each node represent marginal probabilities for alternative ancestral locations.

**Figure 6:**
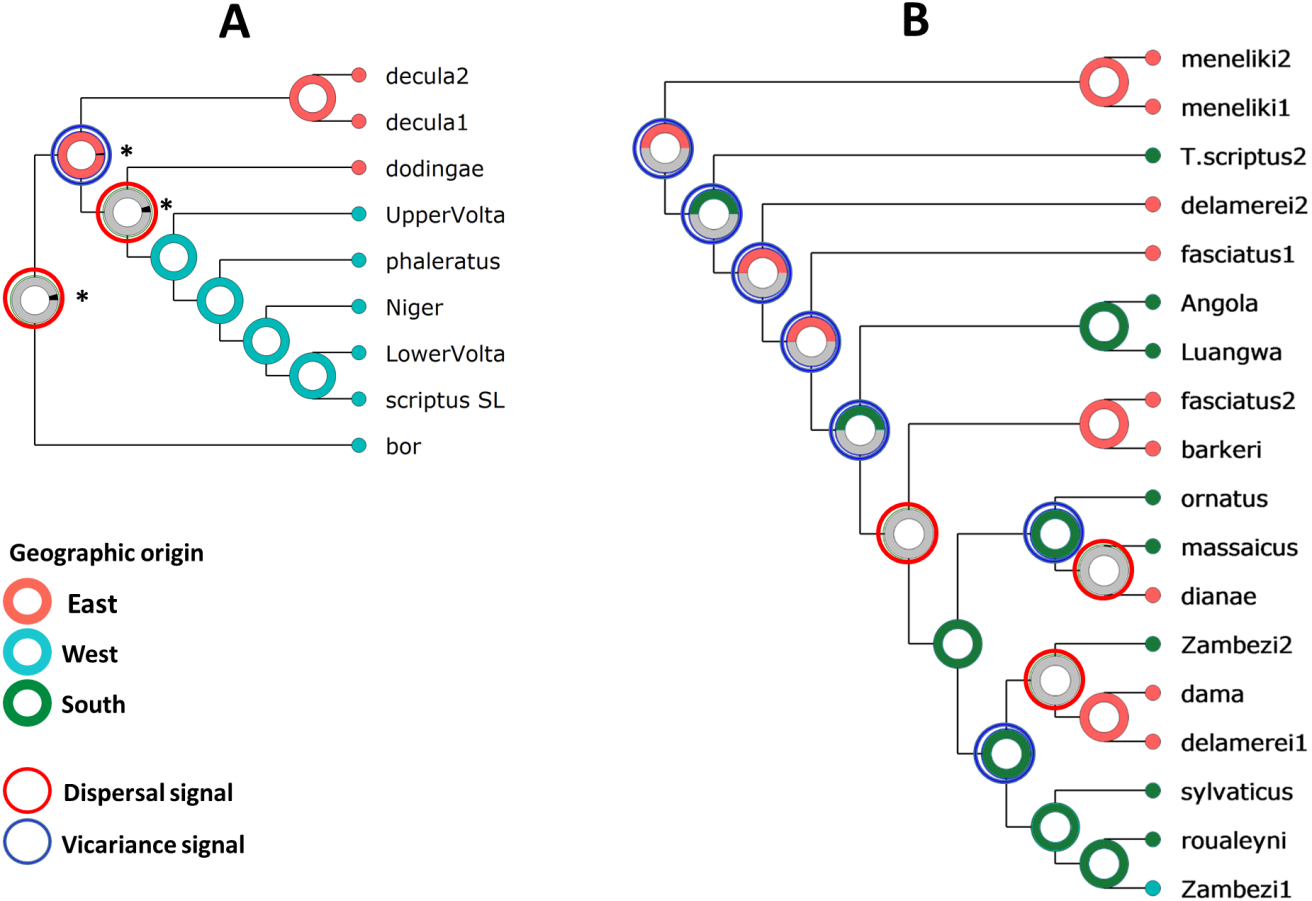
Statistical reconstructions (Pie charts) of ancestral areas based on the S-DIVA analyses of nuclear DNA for Scriptus (A) and Sylvaticus (B). Colour coding follows Fig. 5. Three major distributions were defined as East Africa (E), West Africa (W), southern Africa (S). For each node only the most likely reconstruction was considered and ancestral areas with probability <0.05 were represented by asterisks.

We used both discrete Bayesian phylogeography and statistical dispersal-vicariance approaches to reconstruct patterns of spatial dispersal and the ancestral location for the origin of the species complex. Within Scriptus, both analyses separated a well-supported *T. s. dodingae*-*T. s. decula* clade in the east, from bushbuck inhabiting regions across the Nile and further west (including the Nile bushbuck, *T. s. bor*, Figs. 5A and 6A). Sylvaticus also comprised significant phylogeographic structuring, with Menelik’s bushbuck most divergent, and other more derived lineages separated into eastern and southern groups (Fig. 5B) or into different groups with histories of either dispersal or vicariance (Fig. 6B). Both approaches identified East Africa as the most likely ancestral location for the origin of the bushbuck radiation. From this origin, Bayesian phylogeography invoked dispersal events in a westward direction for Scriptus and in a southward direction for Sylvaticus, both events occurring on either side of the Congo basin. On the other hand, S-DIVA analysis allowed for the possibility of vicariance, rather than dispersal, as an explanation for nuclear DNA spatial branching patterns. According to this analysis, the initial split from an ancestral Scriptus was a westward dispersal of *T. s. bor* into central Africa, followed by vicariance that separated *T. s. decula* from *T. s. dodingae*, and a subsequent secondary dispersal into West Africa. In contrast, the initial stages of the Sylvaticus radiation into southern Africa are all characterized by vicariance events, with dispersal only invoked for more derived lineages around the Great Lakes region.

### Ecological adaptation

MRM analysis revealed that biogeography explained a significant 95% of the nuclear genetic variation within the species complex (Table 4). Taxonomic designation and geographic distance accounted respectively for 88% and 26% of the variation, with the more recent taxonomy of Groves & Grubb (2011) outperforming previously used schemes. Under the conditional influence of isolation by distance, both biogeographic and taxonomic models account for 41% and 65% of the genetic variation respectively.

## Discussion

### Patterns of genetic diversity

Nuclear genetic diversity was moderate across the species complex. However, mtDNA diversity was exceptionally high, with only a handful of studies reflecting similar levels (Arctander, Johansen & Coutellec-Vret, 1999; Smitz et al., 2013). That mtDNA diversity was higher than nuclear DNA is expected given the differences in mutation rates between the two sets of loci (Nei & Kumar, 2000), however, particularly high mtDNA diversity in bushbuck also reflect a Pliocene mtDNA introgression event ( Hassanin et al., 2018), where Scriptus obtained a nyala-like mitochondrial genome. Within each lineage, the higher diversity of Sylvaticus at both nuclear and mtDNA reflects a slightly earlier coalescence time relative to Scriptus (Fig. 3).

### Origins, divergence and secondary contact

Fossil records from the mid-Pliocene (approximately 3.9 Mya) of proto-bushbuck are known from several sites in eastern and southern Africa. *T. scriptus* remains were recovered in Ethiopia (Kalb et al., 1982) and Kenya ( Harris et al., 1988; Leake & Harris, 2003). We observed a more recent diversification of Sylvaticus and Scriptus lineages. Since these fossils predate the estimated divergence within the bushbuck, they suggest a possible ancestral origin from north-east Africa. This is indeed the inference from our Bayesian phylogeography and S-DIVA reconstructions, supporting an origin for the species in East Africa. Until the late Pliocene, east Africa was densely forested habitat (Partridge, Wood & DeMenocal, 1995; Reed, 1997), supporting the idea that ancestral bushbuck were both forest dwelling and used its peculiar harnessed striping pattern as an adaptation for camouflage in closed habitats (Moodley & Bruford, 2007). There is some evidence that striping patterns among other bovids are also associated with living in forest habitat (Stoner, Caro & Graham, 2003).

The past 3–2 Mya has seen a major paleoclimatic shift that led to the expansion of grassland habitats in Africa, consequently inducing a drastic change in ungulate community structure, specifically in north-east Africa (Bobe & Behrensmeyer, 2004;  Hernandez Fernández & Vrba, 2006; Trauth et al., 2007). This also coincided with major geomorphological processes along the Gregory and Albertine Rifts (Vrba, 1995; Reed, 1997). The combination of paleoclimatic shifts and tectonic uplift have shaped the phylogeography of terrestrial African vertebrates (Flagstad et al., 2001; Trauth et al., 2007; Lorenzen et al., 2010; Voelker, Outlaw & Bowie, 2010; Faulkes et al., 2011; Barlow et al., 2013; Jacobs et al., 2013). The Scriptus-Sylvaticus divergence can also be traced back to this time, and their extant distributions on either side of the Rift Valley (Fig. 1) suggest vicariance of the two lineages, on the basis of the major tectonic uplift events along the East African Rift system. Statistical dispersal-vicariance analysis also suggest that a dispersal-only view of branching events within Scriptus and Sylvaticus from their East African origin may be too simplistic. The evolutionary history of Sylvaticus is predominated by vicariance events, which may help explain why phenotypic diversity is higher in this lineage compared to Scriptus, where a history of mainly dispersal was invoked.

**Table 4:** Fitting of bushbuck nuclear DNA genetic distance data against taxonomic, biogeographic, and geographic models.

		**Multivariate matrix regression**				
**Predictors**	**Model**	**df**	**pseudo-F**	**Marginal**	**pseudo-F**	**Conditional**
Taxonomy	All subspecies	25	2.049	0.770	1.886	0.657
	Groves and Grubb	25	15.771	0.881^**^	24.372	0.700^**^
Biogeography	Olson et al.	25	7.893	0.953^*^	0.338	0.414^**^
Geography	Coordinates	25	4.130	0.264^*^	–	–

permutation *P* < 0.05*; <0.01**

Since divergence, Scriptus and Sylvaticus appear to have remained geographically isolated, however, gene flow between the two cannot be discounted. Although mitochondrial and nuclear multilocus haplotypes were not shared between Scriptus and Sylvaticus, the most common allele at nuclear genes PRKC1, SPTBN1 and THY and the mitochondrial 12S rRNA were shared among samples of both lineages. Shared alleles may indicate polymorphisms that were present in an ancestral bushbuck population, but they may also indicate post-divergence gene flow between Scriptus and Sylvaticus. A further analysis with whole genome sequences may yet shed further light on the role of introgression in the evolution of this species complex.

### A stable Pleistocene demographic history

Both bushbuck lineages appear to have been demographically stable through the mid to late Pleistocene (Table 3, Fig. 4), despite most of the diversity within each lineage having evolved during this time. This is a surprising result, as the Pleistocene is known for its dramatic climatic fluctuations. Ungulate population sizes are inherently linked with climate change over evolutionary timescales (Lorenzen et al., 2011), and the distributions of herbivores would presumably have shifted in accordance with vegetation change. In sub-Saharan Africa, Pleistocene population expansions of large mammals such as the kob (Birungi & Arctander, 2000), Jackson’s hartebeest (Flagstad et al., 2001), Cape buffalo (Van Hooft, Groen & Prins, 2002; Smitz et al., 2013), hippopotamus (Okello et al., 2005) and lion (Barnett et al., 2014) tend to corroborate this view. Pleistocene demographic contractions such as that of the brown hyaena (Westbury et al., 2018) occur less commonly among African mammals, with most declines taking place during the Holocene, as observe for drill baboons (Ting et al., 2012) and white rhinoceros (Moodley et al., 2018). Yet, during the same period, bushbuck mitochondrial and nuclear DNA shows little evidence of demographic change since the Scriptus-Sylvaticus divergence. It is possible that bushbuck, being highly adaptable and ubiquitous generalists, are less demographically affected by climatic fluctuations, and that evolutionary change occurs more through vicariance than population size changes.

### Rapid ecological specialization

Demographic stability also appears to be at odds with high levels of variation observed both morphologically and genetically. The extant genetic diversity in both Sylvaticus and Scriptus was generated in the late Pleistocene, <1 Mya, but with most divergences occurring within the last 0.5 Mya. Much of this diversity is reflected in mitochondrial DNA (Fig. 2A), and has been described previously (Moodley & Bruford, 2007). Although, fewer divergence events were identified with nuclear intron sequences, a large proportion of the nuclear sequence diversity could be attributed to biogeography, even when conditioned on geography (Table 4). This lends strong support to the hypothesis that local ecology has helped shape the structure of genetic diversity in this species.

By dating our nuclear tree we were also able to estimate a more realistic timeframe for the onset of divergence events in the species complex, compared to the mtDNA-based timeframes reported by Moodley & Bruford (2007). Within Sylvaticus, Menelik’s bushbuck (*T. s. meneliki*) was first to diverge into cooler habitats of the Ethiopian massif. Larger size, a darker and thicker coat are typical of several mammalian montane forms (egs. Red squirrel, *Paraxerus palliates;* Saola, *Pseudoryx nghetinhensis*). Bergman’s rule predicts an increase in size among colder-adapted species (Bergmann, 1847; Freckleton, Harvey & Pagel, 2003; Clauss et al., 2013), whereas darker and thicker coats help in thermoregulation (Caro, 2005; Clusella-Trullas et al., 2008; Mills & Hes, 1997; Amy & Kunz, 2012). The early differentiation of montane Menelik’s bushbuck, and the more recent evolution of other montane bushbuck (eg. *T. s. barkeri*, *T. s. delamerei*) strengthens evidence for the independent convergence of the montane phenotype among Sylvaticus bushbuck.

The Somali bushbuck (*T. s. fasciatus*) is also large in size and is able to survive deep into the xeric interior of the Horn of Africa along the watercourses of the Wabi Shebelle and the Juba River. This population comprises two paraphyletic mtDNA lineages (Fig. 2A) and independent nuclear lineages (Fig. 2B), suggesting the bushbuck colonized the Somali arid zone through two migration or range expansion events of different coastal bushbuck populations from the south.

Within Scriptus, the Nile-Abyssinian bushbuck (*T. s. bor-T. s. decula*) clade diverged into the more open, drier habitats of the mosaic region on the fringes of the Sahel. This is reflected in phenotype, as most Scriptus populations are strikingly patterned with the typical bushbuck “harness”, striping is reduced in those Scriptus populations in more open habitats such as *T. s. bor*, *T. s. decula* and *T. s. dodingae*. There is also a suggestion of reduced patterning among Sylvaticus bushbuck. Although much less strikingly coloured, individuals in some Sylvaticus populations such as the Chobe bushbuck (*T. s. ornatus*) and the Ituri bushbuck (*T. s. dianae*) may be more heavily patterned with vertical and horizontal stripes and spots. However, such individuals become rarer in populations to the south where habitats are drier and more open. A similar loss of patterning occurs across the north-south range of the plains zebra, which is also suggested to be in response to open drier environments (Rau, 1978; Leonard et al., 2005).

## Conclusions

In the present study, we sequenced mitochondrial and nuclear DNA 27 individuals representing the range of genetically distinct haplogroups previously described within the bushbuck complex. Phylogenetic congruence was observed between mitochondrial and nuclear markers, both identifying two lineages that diverged in the late Pliocene (Scriptus and Sylvaticus), with further diversification into more specialised groupings during the Pleistocene. Although climatic upheaval during the Pleistocene may have promoted one of the more astonishing arrays of phenotypic diversity among mammals in Africa, we do not observe evidence that these changes were effected by decreases in population size (genetic drift). The strong association between genetic diversity and ecological region suggests that the exceptional diversity within the bushbuck complex may have been driven, at least in part, by parapatric speciation.

##  Supplemental Information

10.7717/peerj.6476/supp-1 Data S1 Raw sequences data

## Additional Information and Declarations

### Data Availability

The following information was supplied regarding data availability: The raw sequences data are provided in Data S1.
